# Chemical warfare between fungus-growing ants and their pathogens

**DOI:** 10.1016/j.cbpa.2020.08.001

**Published:** 2020-12

**Authors:** Sibyl F.D. Batey, Claudio Greco, Matthew I. Hutchings, Barrie Wilkinson

**Affiliations:** 1 Department of Molecular Microbiology, John Innes Centre, Norwich, NR4 7UH, United Kingdom; 2 School of Biological Sciences, University of East Anglia, Norwich, NR4 7TU, United Kingdom

**Keywords:** Fungus-growing ants, Mutualism, Antagonism, Specialized metabolites, *Escovopsis*, *Pseudonocardia*, *Streptomyces*, Antimicrobials

## Abstract

Fungus-growing attine ants are under constant threat from fungal pathogens such as the specialized mycoparasite *Escovopsis*, which uses combined physical and chemical attack strategies to prey on the fungal gardens of the ants. In defence, some species assemble protective microbiomes on their exoskeletons that contain antimicrobial-producing Actinobacteria. Underlying this network of mutualistic and antagonistic interactions are an array of chemical signals. *Escovopsis weberi* produces the shearinine terpene-indole alkaloids, which affect ant behaviour, diketopiperazines to combat defensive bacteria, and other small molecules that inhibit the fungal cultivar. *Pseudonocardia* and *Streptomyces* mutualist bacteria produce depsipeptide and polyene macrolide antifungals active against *Escovopsis* spp. The ant nest metabolome is further complicated by competition between defensive bacteria, which produce antibacterials active against even closely related species.

## Highlights

• Specialist fungal pathogens attack the nests of fungus-growing ants.
• Ants form mutualistic relationships with defensive actinomycete bacteria.
• Specialised metabolites underpin these mutualistic and antagonistic interactions.

## Introduction: farmers at war

Ants of the tribe Attini cultivate basidiomycete fungi in a process akin to human agriculture that evolved around 50–60 million years ago [1]. The Attini can be divided according to their fungal cultivars, which include species across the tribe Leucocoprineae. The lower attines tend to have less specialized fungal cultivars, which they feed with dead biomass, including vegetative debris and insect corpses [2]. The higher attines have specialized obligate fungal cultivars which have developed hyphal swellings known as gongylidia that provide a rich source of nutrients for the colony [3]. The leafcutter ants are the most highly derived attines and comprise the genera *Atta* and *Acromyrmex* [2]. They mainly cultivate *Leucoagaricus gongylophorus* [4], a polyploid clone, which is transmitted vertically between nests [5]. The ants actively cut fresh leaf material to feed to their cultivar, which provides the sole food source for the ant larvae [2].

The most significant threat to this mutualistic endeavour comes from specialized fungal pathogens in the genus *Escovopsis*, which have co-evolved with attines and are highly adapted for a mycoparasitic lifestyle [6]. More recently, *Escovopsiodes* have been identified as a further distinct mycoparasitic genus [7]. If left unchecked, *Escovopsis* spp. can overrun a nest leading to devastating colony collapse [8]. The most well-studied species is *Escovopsis weberi* [9], commonly found in the nests of leafcutter ants. The ants carefully groom and weed their fungal gardens to remove *Escovopsis* and other pathogens [2], modifying their hygiene strategy according to the growth stage of the pathogen [10]. Many attines also house and feed defensive bacteria on their cuticles, initially a single vertically transmitted *Pseudonocardia* phylotype transferred to newly eclosed workers and virgin queens from their sisters and/or the fungal cultivar [11]. Other actinomycetes, such as *Streptomyces* species, are horizontally acquired from the environment to form a protective microbiome [12]. These bacteria produce antifungal compounds that are active against *Escovopsis* spp., as well as antibacterials to outcompete other defensive bacteria (Table 1) [13]. Within the leafcutters, there has been an interesting divergence, with only *Acromyrmex* maintaining a protective microbiome, whereas *Atta* rely on their own endogenous chemical defences [14]. However, *Escovopsis* comes armed with a chemical war chest of its own that includes antibacterial, insecticidal and antifungal specialized metabolites that target each component of the ant-microbe symbiosis (Table 1) [15,16].

**Table 1:** Summary of bioactive compounds isolated from fungus-growing ant mutualists and pathogens.

Producer	Ant Species	Compound	Bioactivity	References
***Escovopsis***				
*E. weberi*	*Acromyrmex octospinosus*	Shearinine L (**1**)	- A. *octospinosus* ants chose not to eat **1**-impregnated oat flakes	[15]
*E. weberi*	*Acromyrmex echinatior*	Shearinine D (**2**)	- Adverse effect on *A. echinatior* ant behaviour, lethal at high concentrations. - Active against *Pseudonocardia echinatior* and *Pseudonocardia octospinosus* strains isolated from *A. echinatior* colonies.	[15,16]
*E. weberi* *E. aspergilloides*	*A. echinatior* *Trachomyrmex cornetzi*	Melinacidin IV (**3**)	- Active against *P. echinatior* and *P. octospinosus* strains isolated from *A. echinatior* colonies.	[16]
*E. weberi* *E. aspergilloides*	*A. echinatior & A. octospinosus* *T. cornetzi*	Emodin (**4**)	- Active against *Leucoagaricus gongylophorus* and Streptomyces strains isolated from leafcutter ant nests.	[15,16]
*E. weberi*	*A. echinatior*	Cycloarthropsone (**5**)	- Active against *L. gongylophorus*.	[15]
***Streptomyces***				
S4, Ao10	*A. octospinosus*	Candicidin D (**9**)	- Active against E. weberi but not against *L. gongylophorus*.	[36,38,40]
Ae32_2, S4, Ao10 Av28_2, Av28_3 Av25_1	*A. echinatior* *A. octospinosus* *Acromyrmex volcanus*	Antimycin A1-A4 (**10a-d**)	- Active against *E. weberi*. and *L. gongylophorus*.	[39,40]
ICBG292	*Cyphomyrmex sp.*	Mer-A2026B (**11**) /Piericidin-A1 (**12**)	- Active against different *Escovopsis spp*. - Antileishmanial activity.	[41]
Av25_2	*A. volcanus*	Actinomycin D (**16**) Actinomycin X2 (**17**)	- Active against *Pseudonocardia* and *Streptomyces spp*.	[39]
***Pseudonocardia***				
CC011120-4	*Apterostigma dentigerum*	Dentigerumycin A (**6**)	- Antifungal activity against *E. weberi*	[32]
*P. octospinosus* P1	*A. octospinosus*	Nystatin P1 (**7**)	- Antifungal activity against *E. weberi*	[29,36]
*HH130629-09, HH130630-07*	*Apterostigma sp.*	Selvamicin (**8**)	- Antifungal activity, activity against *E. weberi* not reported	[37]
BCI2	*A. dentigerum*	9-methoxyrebeccamycin (**13**)	- Inhibits other *Pseudonocardia* strains.	[47]
17SE-9	*Trachymyrmex septentrionalis*	GE37468 (**14**)	- Inhibits *Pseudonacardia spp*. from the same ant nest.	[48]
EC080529-01	*A. dentigerum*	6-deoxy-8-O-methylrabelomycin (**15**)	- Antibacterial and antimalarial activity	[49]

**Figure 1:**
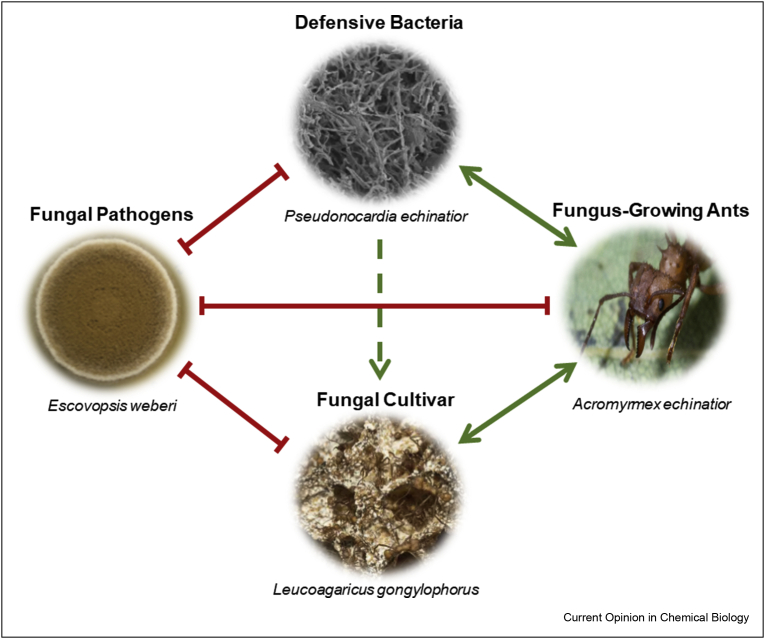
**A network of mutualistic and antagonistic interactions exists in the nests of fungus-growing ants**. Representative examples of each member of the symbiotic network are shown, with *Pseudonocardia* hyphae imaged by scanning electron microscopy (SEM). Red lines indicate antagonistic interactions, green arrows indicate mutualistic interactions and the green dashed arrow indicates indirect mutualism.

Thus, there exists a highly co-evolved, complex network of mutualistic and antagonistic relationships within the nests of fungus-farming ants, Figure 1. Here, we review recent advances in our understanding of the sophisticated attack and defence strategies utilised, focussing on the complex array of chemical signals underpinning each interaction.

## Under attack

### Physical attack

The mechanism of parasitism by *Escovopsis* and *Escovopsioides* species requires the pathogens to have physical contact with the cultivar fungus. Early observations reported that *E. weberi* grows faster in the presence of *L. gongylophorus* and causes hyphal degradation. In addition, different *Escovopsis* isolates vary in the level of selectivity toward the cultivar fungus [17]. Building on this work, Marfetán and colleagues [18] showed that all *E. weberi* strains were, at different levels, virulent towards *L. gongylophorus* and that the most virulent isolates could develop hook-like structures that were used to attach to the fungal cultivar. More recently, several *Escovopsis* species and two *Escovopsioides nivea* strains were reported to inhibit the growth of *L. gongylophorus* in co-cultivation experiments where both the pathogen and the garden fungus had a drastic change in pigmentation [9,19]. In addition, when *Escovopsis* spp. were grown on water agar with or without a small colony of *L. gongylophorus* it was found that the *Escovopsis* spp. grew only on co-cultivation plates and the pathogen grew directly towards *L. gongylophorus* forming hyphal bridges, and outgrew it within 48 h [18].

### Chemical attack

**Figure 2:**
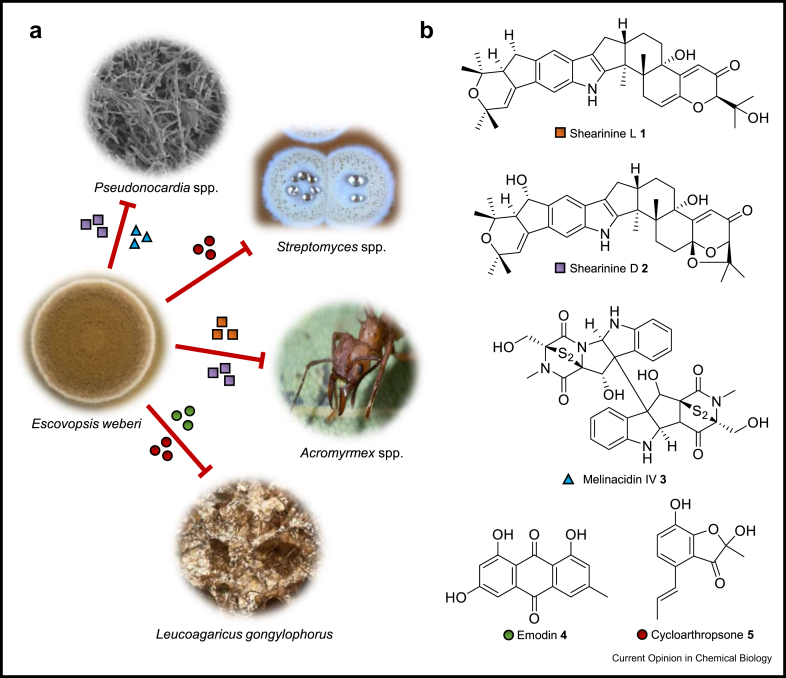
**a) Antagonistic interactions of *Escovopsis weberi* with other organisms.** Representative images of each symbiont are shown with red lines indicating antagonistic interactions. Specialized metabolites are represented with coloured shapes, as shown in **b**. **b) Chemical compounds isolated from *E. weberi*.** Main classes identified thus far are the shearinine terpene-indole alkaloids and the melinacidin epipolythiodiketopiperazines (ETPs), as well as other polyketide metabolites such as cycloarthropsone and the anthraquinone emodin.

While the threat posed by *Escovopsis* species to fungus-growing ants has been well-established, the specialized metabolites it utilises to modulate this interaction have only recently begun to be uncovered, Figure 2. Indirect evidence came from the first genome sequence of *E. weberi* and associated RNA-sequencing data which identified that a biosynthetic gene cluster (BGC) for a polyketide synthase-derived specialized metabolite was upregulated when *E. weberi* and *L. gongylophorus* are co-cultured [9]. Rodrigues and colleagues reported that chemical extracts from *Escovopsis* species and *E. nivea* cultures were able to inhibit the growth of *L. gongylophorus* and that the extracts obtained from co-cultures of pathogen and cultivar were generally more active than extracts from axenic plates of the pathogen [19]. Another recent study identified the production of the terpene-indole alkaloid shearinines by *Escovopsis* sp. TZ49, using a combination of imaging mass spectrometry and MS/MS molecular networking [20]. In subsequent separate studies, shearinine metabolites were shown to be upregulated when *E. weberi* was grown in the presence of *L. gongylophorus* [16], and several shearinine congeners were isolated and characterised from a range of *Escovopsis* strains (six *E. weberi* isolates and one *Escovopsis aspergilloides*) [15,16]. Worker ants learned not to choose oat flakes impregnated with shearinine L (**1**), but there was no obvious effect on waste production or worker mortality in the colony [15]. However, shearinine D (**2**) supplied as a glucose solution in the absence of the fungal cultivar adversely affected the behaviour of worker ants and was ultimately lethal. Compound **2** was also found to be elevated in the tissues of worker ants in a captive colony that suffered a natural *E. weberi* outbreak [16]. This is consistent with the previously documented roles of terpene-indole alkaloids acting as feeding deterrents and modulators of ion channels in various insects [21]. Furthermore, **2** was active against mutualist *Pseudonocardia* strains isolated from *Acromyrmex echinatior* [16] whereas **1** was not active against *L. gongylophorus* or *Pseudonocardia* Ao19 isolated from *Acromyrmex octospinosus* [15]. The variation of responses observed for *Pseudonocardia* species to these different shearinine congeners may be due to their structural variation or could be attributed to the different methods of antimicrobial testing used.

Production of the epipolythiodiketopiperazine (ETP) melinacidin IV (**3**), a known antimicrobial and cytotoxic agent, was also increased when *E. weberi* was co-cultured with *L. gongylophorus* and was shown to inhibit the growth of *A. echinatior* mutualist *Pseudonocardia* species [16]. Two other metabolites were isolated from *E. weberi*, the anthraquinone emodin (**4**) and the polyketide cycloarthropsone (**5**) [15,16], both of which inhibit the growth of the cultivar fungus *L. gongylophorus*. Compound **4** is also produced by *E. aspergilloides* and inhibits the growth of several streptomycete leafcutter ant mutualists [15].

There have been several reports of the isolation of black yeast-like fungi in the order Chaetothyriales from the cuticles of fungus-growing ants, a potential additional player in the symbiotic network [21, 22, 23, 24]. It has been proposed that the black yeasts could indirectly benefit the fungal pathogen as they were shown to inhibit the growth of ant mutualist *Pseudonocardia in vitro* [25]. However, phylogenetic analyses suggest they are not particularly specialized in their interactions with fungus-growing ants [26].

## Defence

### Microbial defences

A highly effective defensive strategy utilised by fungus-growing ants is the assembly of a protective microbiome on the external surface of their exoskeletons, which is dominated by antimicrobial-producing actinobacteria [27]. In addition to protection of the fungal cultivar from *Escovopsis* attack, cuticular microbiomes have been shown to protect worker ants from infection by the entomopathogenic fungus *Metarhizium anisopliae* [28] and are proposed to help shape the cuticular microbiome by excluding non-mutualist bacteria [29]. The presence of the cuticular microbiome may also serve as a physical barrier against fungal infection [30].

The most well-studied bacterial symbionts belong to the *Pseudonocardia* genus, and many attines vertically transmit a single strain of *Pseudonocardia* [12]. The bacteria grow in and around the openings of specialized crypts on the ant cuticle and are proposed to feed on secretions from subcuticular glands [27]. The presence of *Pseudonocardia* species greatly improves the suppression of *E. weberi* infections in leafcutter colonies [30]. The bioactivity of mutualistic *Pseudonocardia* strains against *Escovopsis* spp. has been recapitulated *in vitro* [31], and several antifungal compounds have been identified.

**Figure 3:**
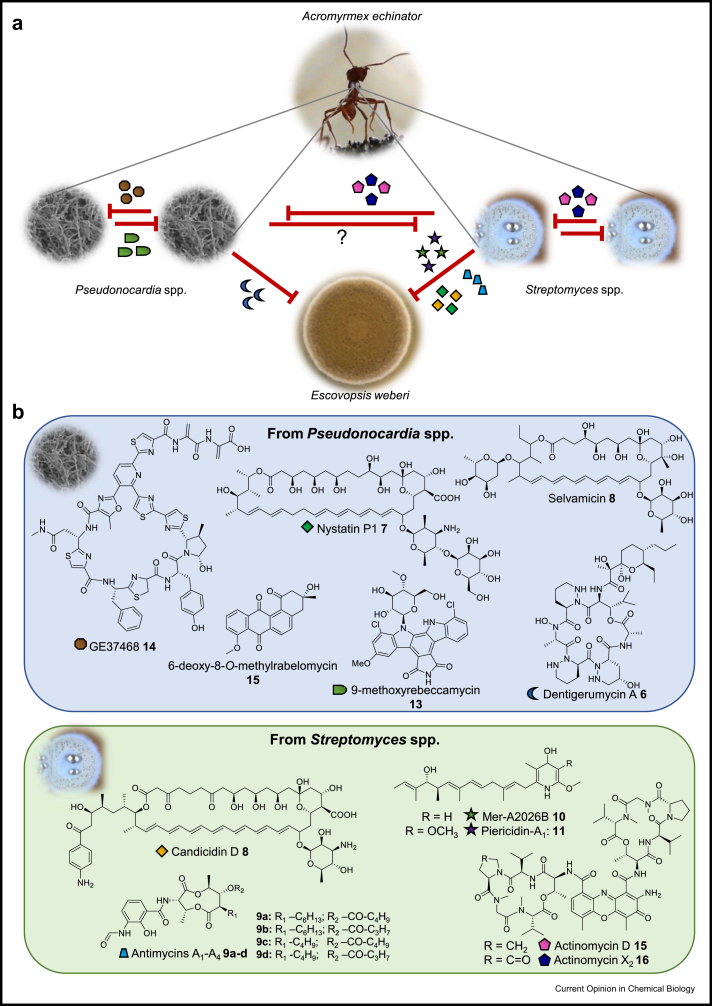
**a) Interactions between defensive Actinobacteria and *Escovopsis weberi*.** The protective microbiome is visible as a white coating on the integument of an *Acromyrmex* ant. Representative images of each symbiont are shown with red lines indicating antagonistic interactions. Compounds are produced by mutualistic bacteria as defence/attack against both the fungal pathogen and competitors. Specialized metabolites are represented with coloured shapes according to **b**. Production of compounds active against streptomycete bacteria by *Psuedonocardia* spp. has not been reported. **b) Specialized metabolites produced by mutualistic bacteria.** The main classes are non-ribosomal peptides (NRPs), such as the depsipeptides, which include dentigerumycin A **6** and the actinomycins **16** and **17**. The antifungal polyene macrolides include nystatin P1 **7**, candicidin D **9** and selvamicin **8**, the latter having an apparently novel mode of action. The activity of **8** and 6-deoxy-8-*O*-methylrabelomycin **15** against species from the nests of fungus-growing ants has not been reported.

*Pseudonocardia* produce two main classes of antifungal molecules: cyclic depsipeptides and polyene macrolides, Figure 3. The cyclic piperazine-containing depsipeptide dentigerumycin A (6) is produced by a *Pseudonocardia* strain isolated from the lower attine *Apterostigma dentigerum*. Dentigerumycin A **6** is of mixed polyketide/non-ribosomal peptide origin and is active against *Escovopsis* pathogens but not the fungal cultivar [32]. Interestingly, dentigerumycin analogues were also identified in *Macrotermes* fungus-growing termites [33], and the closely related gerumycins were isolated from another *A. dentigerum*-derived *Pseudonocardia* species, as well as strains associated with the higher attine *Trachymyrmex cornetzi*, but these did not show antifungal activity against *Escovopsis* species [34].

The other main type of compounds are polyketide polyene macrolides, a well-known class of antifungal that includes the clinically important drugs nystatin A1 and amphotericin B, which act by binding ergosterol [35]. A novel polyene antifungal named nystatin P1 (**7**) was isolated from the *A. octospinosus* derived *Pseudonocardia octospinosus* P1 strain. While the structure of **7** has not been fully elucidated, MS/MS and BGC analysis strongly suggest that it has an additional hexose compared to nystatin A1, likely d-mannose linked to the d-mycosamine moiety via a β-1,4 linkage [29,36]. Selvamicin (**8**) is another novel polyene macrolide, identified from a *Pseudonocardia* strain associated with *A. dentigerum*. It exhibits activity against a range of fungal bioindicator strains; however, its activity against *Escovopsis* has not been reported. The unusual 4-O-methyldigitoxose glycosylation and the lack of charged groups (Figure 3) makes **8** distinct from other antifungal polyene compounds, and as it showed no evidence of ergosterol binding, it appears to have a novel mechanism of action [37].

Other Actinobacteria have also been recovered from the exoskeletons of fungus-growing ants, most notably *Streptomyces* species. The production of the antifungals candicidin D (**9**) and antimycin A_1_-A_4_ (**10a**-**d**) has been observed for *Streptomyces* species derived from *Acromyrmex* nests [36,38, 39, 40]. Interestingly, **9** was highly active against *E. weberi* but not the *L. gongylophorus* cultivar [38], whereas **10a-d** compounds showed more generalised activity against both fungal strains [39]. More recently, Streptomyces species isolated from the exoskeletons of Cyphomyrmex and *Acromyrmex rugosus* workers were found to produce the monohydroxypyridines Mer-A2026B (**11**) and piericidin A (**12**), along with the ionophores nigericin and dynactin, all of which inhibited the growth of various *Escovopsis* spp. in challenge experiments on agar. Interestingly, these compounds were also found to have antileishmanial activity, the first report of antiprotozoal activity by ant-nest compounds [41].

Beyond *Pseudonocardia* and *Streptomyces* species, yeasts isolated from *Atta texana* nests inhibited the growth of *E. weberi*, along with generalist pathogenic and entomopathogenic fungi [42]. *Bulkholderia* species have also been found in fungus-growing ant nests, and one strain isolated from an *Atta sexdens rubropilosa* colony inhibited the growth of *E. weberi* and other fungal pathogens, but not *L. gongylophorus* [43]. These reports hint at the much more complex interplay of different species in the nests of fungus growing ants, beyond the well-studied mutualists and pathogens.

The fungal-symbionts themselves have been implicated in defence against *Escovopsis*; however, reports of their antifungal capabilities have been variable [14]. The unique yeast cultivar maintained by the lower attine *Cyphomyrmex minutus* produced antifungal diketopiperazines [44]. More recently, leafcutter cultivars were shown to inhibit *Escovopsis* strains from lower attine ants, such as *Apterostigma* [45].

#### Infighting

As well as producing antifungals to suppress host pathogens, defensive symbionts produce antibacterials to outcompete microbial competitors [29]. Indeed, antagonism between *Pseudonocardia* strains is common, and in challenge experiments pairing strains from across the broader phylogeny, closely related strains were able to inhibit each other [46]. The indolocarbazole 9-methoxyrebeccamycin (**13**) was produced by *Pseudonocardia* isolated from *A. dentigerum* and inhibited other *Pseudonocardia* strains isolated from the same region [47]. Recently, the thiopeptide GE37468 (**14**) was isolated from *Pseudonocardia* associated with *Trachymyrmex septentrionalis* ants and shown to inhibit other *Pseudonocardia* strains isolated from *Trachymyrmex* nests [48]. Another *A. dentigerum*-derived *Pseudonocardia* strain produced the angucyclines 6-deoxy-8-*O*-methylrabelomycin (**15**) and X-14881, and the glycosylated pseudonocardones. The non-glycosylated compounds showed some antibacterial and antimalarial activity, but their role in ant nests is yet to be established [49]. Streptomycetes associated with fungus-growing ants also produce antibacterials, most notably actinomycin D (**16**) and actinomycin X_2_ (**17**), which inhibited both *Pseudonocardia* and other *Streptomyces* species and acted synergistically with **10a**-**d** compounds. Valinomycins were also produced by several *Streptomyces* spp.; however, these were not active against the ant-nest species tested [39].

### Ant defences

In marked contrast to the *Acromyrmex* leafcutters, *Atta* ants lack cuticular actinomycete cultures. Instead, they rely on phenylacetic acid secretions from their metapleural glands to fend off fungal pathogens. Although phenylacetic acid is also active against generalist fungal pathogens, *E. weberi* appears to be particularly vulnerable, with strains isolated from lower attine ant genera being more susceptible than those from higher attines [50]. The reason for the divergence in disease management strategies for these close relatives remains unclear, as do the comparative benefits of chemical *versus* ‘biological’ pest control. On the face of it, the capacity to recruit bacterial symbionts would appear to offer broader evolutionary adaptability; however, the limited evidence of natural *Escovopsis* outbreaks suggests that while they are more widespread in *Atta* colonies, they lead to colony collapse far less frequently than in *Acromyrmex* nests [50].

A range of aldehyde, ketone and alcohol volatiles detected in metapleural gland secretions of *Apterostigma*, *Acromyrmex* and *Atta* have been shown to have strong antibacterial and antifungal activity [51,52]. These compounds may constitute a further, more generalist line of defence in the nest.

### Defence recruitment

Understanding the role of the *Streptomyces* strains commonly isolated from attine cuticles is a crucial next step. One intriguing hypothesis proposes that the ants specifically recruit or ‘screen in’ antibiotic-producing actinomycetes such as *Streptomyces* species while keeping other bacteria out [12,53]. They achieve this by providing public resources to create a competitive environment attractive for bacterial colonisation, and then use their vertically transmitted *Pseudonocardia* mutualist strain to create a demanding environment in which only antibiotic-producers can colonise and survive. These *Pseudonocardia* strains are known to make broad-spectrum antibacterial molecules that inhibit most unicellular bacteria, but they do not inhibit *Streptomyces* species, which themselves make multiple antimicrobials and carry multiple antibiotic resistance genes [29]. The result is an ant cuticle dominated by *Pseudonocardia* and *Streptomyces* species, which make several antibacterial and antifungal compounds that are useful to the ants [54].

## Secret weapons

Genome sequencing of bacterial mutualists isolated from fungus-growing ants has shed light on their untapped biosynthetic potential. Analysis of *Pseudonocardia* strains associated with *A. echinatior*, which split into two phylotypes Ps1 (species name *P. octospinosus*) and Ps2 (*Pseudonocardia echinatior*), showed that Ps2 strains have the potential to produce new nystatin derivatives that arise from a novel 3-amino-5-hydroxybenzoic acid biosynthetic starter unit and encode several bacteriocins which may be involved in inter-species competition [29]. Recent population genomic analyses of nearly 50 *Pseudonocardia* strains isolated from *Apterostigma* ants revealed 27 BGC families, including lassopeptides, siderophores, and terpenes [55,56].

Likewise, although *E. weberi* has a reduced genome size consistent with its parasitic lifestyle, genome sequencing reveals the biosynthetic capabilities of *Escovopsis* strains go well beyond what has been observed in the laboratory, thus far. In *E. weberi* isolated from *Atta cephalotes* 17 putative BGCs were identified [8]. A further five *E. weberi* strains isolated from *Acromyrmex* and *Atta* colonies contained 20–23 putative BGCs each, and in common with the previously sequenced strain, these mainly comprised terpene, type I polyketide synthase and non-ribosomal peptide synthetase BGCs [16].

## Conclusions

A multipartite web of mutualistic and antagonistic interactions exists between the symbionts in fungus-growing ant nests [14]. These ancient systems offer a gateway to a wealth of chemical diversity created by a 50 million-year-old arms race and provide tractable models for understanding the functions of specialized metabolites in nature. While the major players are well-established, recent work suggests that these represent only a fraction of the many microbes present in these colonies, and distinguishing symbionts from opportunistic environmental microbes will be important to develop a full understanding of the interactions at play. These symbiotic interactions are widespread in the plant and animal kingdoms and are all likely driven by chemical communication and chemical warfare. For example, a Kenyan fungus growing plant-ant system is already proving to be an exciting resource for the discovery of new chemical diversity [57,58]. Understanding the roles and regulation of microbial specialized metabolites in their natural habitats is crucial if we are to unlock and discover the vast range of activities encoded by these organisms.

## Role of the funding source

This work was supported by the 10.13039/501100000268Biotechnology and Biological Sciences Research Council (BBSRC) via Institute Strategic Program Project BBS/E/J/00PR9791 to the John Innes Centre, 10.13039/501100000268BBSRC responsive mode grants BB/S009000/1 (to BW) and BB/S00811X/1 (to MIH) and 10.13039/501100000270Natural Environment Research Council grants NE/J01074X/1 and NE/M015033/1 (to MIH) and NE/M014657/1 (to BW).

## Declaration of competing interest

The authors declare that they have no known competing financial interests or personal relationships that could have appeared to influence the work reported in this paper.
